# Salivary sex hormones in adolescent females with trichotillomania^☆^

**DOI:** 10.1016/j.psychres.2018.05.012

**Published:** 2018-07

**Authors:** Jon E. Grant, Samuel R. Chamberlain

**Affiliations:** a Department of Psychiatry & Behavioral Neuroscience, University of Chicago, United States; b Department of Psychiatry, University of Cambridge, UK; c Cambridge and Peterborough NHS Foundation Trust, UK

**Keywords:** Trichotillomania, Sex hormones, Progesterone, Estradiol, Testosterone

## Abstract

Trichotillomania is several times more common in women and has peak onset around puberty. The role of sex hormones, however, has received little research. 11 adolescent girls with trichotillomania, post-menarche and not taking birth control, were examined on a variety of clinical measures. Participants provided saliva samples for analysis of estradiol, progesterone, and testosterone levels. Lower progesterone was associated with more severe symptoms and lower levels of all hormones were associated with worse overall functioning. Adolescents with trichotillomania exhibit a range of hormone levels but that lower levels of certain hormones may have important clinical associations.

## Highlights

• Trichotillomania is a disorder largely affecting females and has its onset during puberty for the majority of people.
• Little research has examined the role of hormones in trichotillomania.
• Lower progesterone was associated with more severe symptoms and lower levels of all hormones were associated with worse overall functioning.

## 1 Introduction

Trichotillomania is a functionally impairing and under-recognized psychiatric condition in which individuals repeatedly pull out their own hair (American Psychiatric Association, 2013). The disorder appears to be overwhelmingly female (a recent study of 462 participants found 94% were female) (Grant et al., 2016) and generally has its onset during puberty (10–13 years) (Christenson, 1995, Cohen et al., 1995, Szepietowski et al., 2009, Grant and Chamberlain, 2016). Although described in the medical literature for almost two centuries, trichotillomania remains poorly understood with limited data regarding its pathophysiology (Grant and Chamberlain, 2016, Christenson and Mansueto, 1999). Trichotillomania constitutes a relatively specific type of behavior, characterized in terms of excessive grooming, which also occurs in other animal species. As such, across psychiatric disorders, the biological substrates of trichotillomania may be particularly well suited to translational modeling (D'Angelo et al., 2014).

There are several lines of evidence to suggest that hormonal factors (such as adrenocorticotropic hormone, estrogen, and progesterone) may be involved in the manifestation of trichotillomania, in addition to this being implied by its female preponderance and typically pubertal onset. Hormonal factors are able to induce grooming in preclinical models, and grooming under normal conditions is contingent on the integrity of dopamine and/or opiate receptors indirectly modulated by such hormones (Traber et al., 1988). Central administration of adrenocorticotropic hormone (ACTH) has been found to induce grooming activities across different animal species (Traber et al., 1988). In animal models of compulsive behavior (such as nesting and marble burying), acute reduction of estrogen/progesterone by ovarectomy leads to increases in compulsive behavior, which are ameliorated by administering these hormones exogenously (Flaisher-Grinberg et al., 2009; Fernandez-Guasti et al., 2006; Mitra et al., 2016). In the case of adults with trichotillomania, Christenson and colleagues noted that 20% of a sample of 56 adults with trichotillomania reported symptom fluctuation depending on time in the ovarian cycle (Christenson et al., 1991). Keuthen and colleagues examined the topic in greater detail and found that 53.3% of 45 participants reported that menstruation affected their pulling with the week before menstruation as the time for symptom exacerbation (Keuthen et al., 1997). In addition, the week before menstruation was also associated with stronger urges to pull and less of an ability to control the pulling (Keuthen et al., 1997). Finally, gonadal hormones have been implicated as playing some role in both Tourette's syndrome and obsessive compulsive disorder (Dillon and Brooks, 1992, Schwabe and Konkol, 1992, Neziroglu et al., 1992), disorders that have phenomenological similarities and comorbid overlap with trichotillomania.

Although these limited studies shed some light on the topic of the possible role of sex hormones in trichotillomania, many unanswered questions remain. Understanding the role of hormones in repetitive behaviors such as trichotillomania may allow for a greater understanding of the pathophysiology and therefore treatment. Based on the extant literature, we hypothesized that sex hormones would be abnormal in young woman with trichotillomania (i.e. lower progesterone levels, higher estradiol levels, and normal testosterone levels). By examining the relationship between hormones and trichotillomania we hope to determine a possible deeper understanding of the biology of this potentially disabling disorder.

## 2 Methods

### 2.1 Subjects

Data from 11 female participants with primary trichotillomania were recruited for a study examining the possible association between their menstrual cycle and trichotillomania symptoms. Study procedures were carried out in accordance with the Declaration of Helsinki. The Institutional Review Board at the University of Chicago approved the study. Adolescents provided written assent after parent consent was obtained.

All subjects had a current DSM-5 primary diagnosis of trichotillomania (APA, 2013). Other inclusion criteria included age 10 to 18 years, post-menarche, no history of taking birth control, and the ability to be interviewed in person. Exclusion criteria included current or past use of birth control, or prior/current diagnosis of bipolar disorder or psychosis.

### 2.2 Assessments

Adolescents with a primary diagnosis of trichotillomania were examined using a semi-structured interview focusing on the clinical features of trichotillomania. Each participant provided their history of psychiatric disorders. Participants also provided a saliva sample, which was stored at −20°Celsius until analysis. Saliva levels of estradiol, progesterone, and testosterone were quantified using standard radioimmunological methodology by Salimetrics.

Severity of TTM, psychosocial dysfunction, and quality of life were assessed with the following measures:

The *Massachusetts General Hospital Hair Pulling Scale (MGH*-*HPS)* (Keuthen et al., 1995). The MGH-HPS is a valid and reliable seven-item, self-report scale that rates urges to pull hair, actual amount of pulling, perceived control over behavior, and distress associated with hair pulling over the preceding seven days.

The *NIMH Trichotillomania Severity Scale (NIMH-TSS)* (Swedo et al., 1989). The NIMH scale is a five-item, clinician-administered scale that rates hair-pulling symptoms during the past week. The items assess pulling frequency (both on the previous day and during the past week), urge intensity, urge resistance, subjective distress, and interference with daily activities.

*Sheehan Disability Scale (SDS)* (Sheehan, 1983). The SDS is a valid and reliable scale that evaluates psychosocial dysfunction in three domains: work/school, social life, and home/family life.

### 2.3 Data analysis

Demographic, clinical, and salivary hormone data were presented in summary form. Relationships between clinical measures (NIMH-TSS, MGH-HPS, SDS total scores) and salivary hormone levels (estradiol, progesterone, testosterone) were explored using correlation tests (Spearman's r). Statistical significance was defined as *p* < 0.05 two-tailed.

## 3 Results

Eleven adolescent females had a mean age of 13.9 ± SD 1.4 [range 11–16] years. Of the 11 females, nine were Caucasian, one was African American, and one was of mixed race. The mean scores on the NIMH-TSS and on the MGH-HPS were 9.1 (±SD 4.6) and 14.4 (±5.5), respectively, reflective of moderate symptom severity. Of the 11 participants, 3 (27.3%) had co-occurring skin picking disorder and 2 (18.2%) major depressive disorder. Two (18.2%) were taking sertraline and one (9.1%) was taking fluoxetine.

Saliva testing showed the following levels (all expressed in mean ±SD pg/mL, with the current study range shown in square brackets): estradiol [3.24 ± 2.75; range 1.57–10.94], progesterone [212.08 ± 165.92; range 19.75–646.42], and testosterone [72.10 ± 22.00; range 26.13–103.94]. Compared to normative data from healthy similarly aged females elsewhere, the mean hormone levels in the TTM sample had the following Z scores: estradiol z = −0.52, progesterone z = +6.42, testosterone z = +3.58 (Op de Macks et al., 2011).

Correlation analysis showed that lower progesterone levels were significantly associated with worse symptom severity on the NIMH-TSS (r = −0.66, p = 0.039). Furthermore, lower levels of all three hormones were each associated with significantly worse disability on the SDS (estradiol r = −0.83, p = 0.006, progesterone r = −0.76, p = 0.017, testosterone r = −0.75, p = 0.021). The other correlation analyses were non-significant (all *p* > 0.3).

The achieved sample size had >80% power to detect a significant correlation with a large effect size of rho = 0.7 or higher, at alpha = 0.05, two-tailed.

## 4 Discussion

In this study of adolescent girls with trichotillomania, the first to examine salivary sex hormone levels, we found that lower progesterone was associated with worse hair pulling severity (NIMH Trichotillomania Symptom Severity Scale, NIMH-TSS), and that lower levels of all three hormones were associated with greater psychosocial dysfunction (Sheehan Disability Scale, SDS). Progesterone is believed to modulate the adaptive response to stress, mainly through the effect of its neurosteroid metabolite allopregnanolone on GABAA receptor activity (Wirth, 2011). Other studies have reported increases in progesterone in women after emotion-arousing stimuli or after stress (Wirth et al., 2007, Childs et al., 2010). Stress-induced increases in progesterone have also been reported in animal studies (Barbaccia et al., 1996). The study did not include its own reference norms, but there was some evidence that trichotillomania was associated with relatively low mean estradiol levels (medium effect size), and unusually high testosterone and progesterone (very large effect sizes), compared to norms published elsewhere for similar aged females.

The finding that lower levels of all three examined hormones (estradiol, progesterone, and testosterone) were associated with higher disability, and also that lower levels of progesterone in particular correlated with worse symptoms, implicates dysregulation of these hormone systems in the pathophysiology of trichotillomania. Virtually all studies of trichotillomania to date (examining neurobiology and treatment; as well as animal modeling) have not assessed hormone levels. The mechanisms through which hormone levels appear to influence symptoms and functioning in people with trichotillomania merits future study. Because testosterone and progesterone appeared unusually high, and yet lower levels of both were associated with worse symptoms, this raises the prospect of possible compensatory biological mechanisms and/or a higher baseline level due possibly to chronic stress responses from an early age (Wirth, 2011).

This study has several limitations. First, due to small sample size, it is unclear how generalizable our results are to the wider population of females with trichotillomania in the community. Also, the study was only powered to detect correlations with large effect size, and due to the small sample size the psychometric properties of the clinical scales were not evaluated (though they have been examined in previous literature) and we were unable to examine effects of comorbidities on the results. Second, this research used correlational analysis without a matched control group. This of course may affect interpretations of the absolute values we have reported but not the correlations with symptoms or dysfunction. Third, polypeptide hormones such luteinizing hormone, follicle-stimulating hormone and gonadotropin-releasing hormone were not examine. Although these hormones are not generally regarded as sex hormones, they interact with and influence the sex hormones. Finally, the samples were one time values and not reported in conjunction with the menstrual cycle for each participant. Despite these limitations, the study sample inclusion/exclusion criteria were fairly broad, and the study used valid objective measures of hormones.

In conclusion, these results demonstrate that lower levels of key sex-axis hormones (estradiol, progesterone, and testosterone) are associated with higher disability in adolescent females with trichotillomania, and with higher symptoms in the case of progesterone in particular. We highlight the need for future studies incorporating such measures, including those conducted in humans and in translational models. If these findings are validated in larger studies, they may suggest new therapeutic directions.

## Appendix Supplementary materials

Image, application 1
